# Myostatin in the Pathophysiology of Skeletal Muscle

**DOI:** 10.2174/138920207783591672

**Published:** 2007-11

**Authors:** Gilles Carnac, Barbara Vernus, Anne Bonnieu

**Affiliations:** 1 INRA, UMR 866-Différenciation Cellulaire et Croissance, 34060 Montpellier Cedex 1, France; 2 INSERM, ERI 25-Muscle et Pathologies, Hôpital Arnaud de Villeneuve, Bât. A Craste de Paulet, 34295 Montpellier Cedex 5, France

## Abstract

Myostatin is an endogenous, negative regulator of muscle growth determining both muscle fiber number and size. The myostatin pathway is conserved across diverse species ranging from zebrafish to humans. Experimental models of muscle growth and regeneration have implicated myostatin as an important mediator of catabolic pathways in muscle cells. Inhibition of this pathway has emerged as a promising therapy for muscle wasting. Here we discuss the recent developments and the controversies in myostatin research, focusing on the molecular and cellular mechanisms underlying the actions of myostatin on skeletal muscle and the potential therapeutic role of myostatin on muscle-related disorders.

## MYOSTATIN PROCESSING AND SIGNAL TRANSDUCTION

Myostatin, also known as GDF8 (growth differentiation factor 8), belongs to the transforming growth factor-β superfamily of secreted growth and differentiation factors [1]. In common with the TGF-β family, myostatin is synthesized as a precursor protein consisting of a signal sequence, an inactive/ inhibitory N-terminal propeptide region and an invariant Arg-X-X-Arg proteolytic cleavage site, followed by a C-terminal domain which dimerizes to form the active/mature molecule [1, 2]. After cleavage of the signal sequence and proteolytic processing, the mature C-terminal dimer remains associated with the propeptide *via *non-covalent forces creating a latent complex from which myostatin must be released to elicit its biological activity [3-6]. Zimmers and coworkers [6] have shown that this latent complex circulates in the blood and can be activated by acid treatment. *In vitro*, latent myostatin activation can also been observed upon proteolytic degradation of the propeptide by a metalloprotease of the bone morphogenetic protein-1/tolloid (BMP1/TLD) family [5]. However, the physiological relevance of such a mechanism is not yet established. 

Although the mechanism of activation of the latent myostatin complex remains to be determined, the bioavalaibility and the function of myostatin appear to be dependent of a network of protein interactions. Indeed, in addition to the propeptide, several other proteins have been identified as inhibitory binding proteins of myostatin. Through a number of yeast two-hybrid system experiments, four binding partners of myostatin (follistatin, hSGT, Titin cap and decorin) have been defined in muscle [7-10] whereas two follistatin related proteins FLRG and GASP have be found to be complexed with myostatin in serum [11, 12]. These proteins are able to negatively regulate myostatin activation, secretion or receptor binding. The effects of these myostatin binding proteins are summarized in Table **1** [2, 7-17]. Interestingly, three of these proteins, the myostatin propeptide, follistatin and FLRG have been shown to be capable of increasing muscle mass when expressed as a transgene in skeletal muscle of wild type mice (Table **1**) [13-15]; a phenotype comparable to those seen in myostatin knock-out mice [1]. However, the increases in muscle mass in the follistatin transgenics are significantly greater than the increases in the myostatin null mice [13]. Furthermore, when the follistatin transgene is combined with a myostatin null mutation, the two phenotypes appear additive as the increase in muscle mass is quadrupled [15]. Such findings indicate that other ligands cooperate with myostatin to control muscle growth and underscore the need to determine the identity of such ligands to elaborate the most effective strategy for manipulating this signalling pathway. 

Although not fully characterized, the signalling pathway and mechanism of myostatin action appear similar to those exhibited by members of the TGF-β superfamily. The mature myostatin peptide binds to one of the two activin type II receptors (ActRIIB to a greater degree than ActRIIA), which recruits, phosphorylates and thereby activates the activin type I receptor propagating signals along the Smad pathway [13, 18]. Myostatin acts through the receptor-associated proteins Smad2 and Smad3 [13, 19, 20]. Phosphorylated Smad2 and Smad3 form heterodimeric complex with the common mediator Smad4. These activated smad proteins function as the key intracellular mediators of signalling for myostatin as they translocate into the nucleus, and activate the transcription of the target genes through interaction with DNA and other nuclear factors [21, 22]. Interestingly, the inhibitory Smad7 protein is implicated in myostatin signalling. Myostatin signalling induces the inhibitory Smad7 protein which in turn abrogates the canonical myostatin signalling pathway, establishing a negative feedback mechanism [23, 24]. 

Like TGF-β family members, myostatin also induces the activation of various MAPK cascades. Philip and coworkers [25] established that myostatin activates the p38 MAPK through the TAK1-MKK6 pathway, independently from SMAD activation. The authors also show that the p38 MAPK functions cooperatively in myostatin-induced, SMAD mediated transcription. It is however unclear how these two pathways - the Smad-mediated pathway and - the TAK1-p38 MAPK pathway communicate but the authors suggest that the type I receptor might be a common upstream signalling component for these pathways [25]. Recent evidence from Yang and co-workers [26] described the involvement of Erk1/2 MAPK cascade in myostatin signalling. Their results suggest that myostatin is capable of activating Erk1/2 MAPK pathway through Ras, a relative upstream intracellular regulator of Erk1/2. Work by this same group has also demonstrated the role of the PI3K/Akt/GSK-3β pathway in myostatin signalling transduction during myogenesis [27] (see below). Finally, another subfamily of the MAPK superfamily, the c-Jun N-terminal kinase (JNK) signalling pathway, is also involved in myostatin signalling [28]. 

Taken together, these results demonstrate that myostatin signalling can recruit various intracellular signalling cascades and suggest that myostatin signalling might be regulated by cross-talks with tyrosine kinases-linked growth receptor signalling pathways and/or other pathways that remain to be elucidated. 

## MYOSTATIN, A FACTOR CONTROLLING MUSCLE SIZE

Muscle size depends on fiber number and fiber size. The number of fibers is usually set during development while their size continously adapts during the entire lifetime depending on activity, nutrition, diseases and aging. In the last decade myostatin has emerged as a key maintenance regulator of muscle mass. The role of myostatin in muscle comes from the phenotype of myostatin-deficient animals. Myostatin was first found to regulate muscle mass in mice from which the gene encoding myostatin has been knocked-out. The resulting “mighty mice” displayed muscle overgrowth due to both hyperplasia (increased number of muscle fibers) and hypertrophy (increased size of individual muscle fibers) [1]. These effects on muscle mass are persistent throughout the life of the animals. Subsequently, the fundamental role of myostatin in adult muscle homeostasis is demonstrated by the increased muscle mass of mice having a postnatal deletion of the myostatin gene or of adult mice injected with various inhibitors of myostatin [5, 29-31]. Therefore, myostatin appears to act at two major points during muscle development; at the level of fiber number during muscle embryogenesis and the level of fiber growth in the adult. 

The function of myostatin appears to have been conserved accross diverse species as indicated by the doubling of muscle mass in cattle, sheep, dog and human carrying natural mutations in the myostatin gene [1, 32-36]. In support of this, myostatin sequence is highly conserved through evolution, among species ranging from zebrafish to humans [1]. Thus, the discovery that myostatin acts as a negative regulator of muscle growth has led to intense scrutiny of the possibility that myostatin inhibition might be useful in treatment of muscle-wasting-related disorders. Accordingly, genetic inhibition of myostatin function or myostatin blockade by several agents (including myostatin antibodies, myostatin propeptide, follistatin-related proteins, soluble type II myostatin receptor) have been shown to be effective in increasing muscle mass and reducing disease severity in the *mdx* mouse model of Duchenne muscular dystrophy, in the caveolin 3 deficient model of limb-girdle muscular dystrophy 1C (LGMD1C) and in two rodent models of amyotrophic lateral sclerosis [19, 37-40]. Although myostatin does not correct the primary defects in muscle dystrophy, it can lessen the severity of the disease phenotype. In contrast, loss of myostatin activity in the *dy^w^/dy^w^* mouse model of laminin-deficient congenital muscular dystrophy, did not ameliorate the muscle pathology but increased postnatal lethality [41]. With respect to the therapeutic benefit of myostatin inhibition, Parsons *et al. *[42] reported that elimination of myostatin can improve the dystrophic phenotype in mice nullizygous for δ -sarcoglycan (*scgd^-/-^*) (a model of human limb-girdle muscular dystrophy referred as LGMD2F). However, this effect is obtained only in young dystrophic mice but not in older ones. In addition, their results also showed that the effectiveness of myostatin blockade could be dependent on specific muscle groups studied. Taken together these studies indicate that myostatin inhibition may not be beneficial in all dystrophic contexts. This is supported by data showing that AAV-mediated delivery of a mutated myostatin propeptide in the muscles of animal models of two limb-girdle muscular dystrophies (LGMD2A caused by mutations in the calpain gene and LGMD2D caused by mutations in the α -sarcoglycan gene) leads to different outcomes: it ameliorates calpain 3 but not α -sarcoglycan deficiency [43]. Therefore, it will be important for investigating the myopathies that are susceptible to myostatin antagonism, and, conversely, for identifying the stages of the disease at which the myostatin blockade is operative.

There is also controversy about the potential improvement of muscle strength in response to myostatin inhibition. Using front paw grip strength, several studies have reported an improvement of force generation in myostatin knock-out mice as well as in wild type mice treated with antibody anti-myostatin or immunized with a DNA vaccine against myostatin [31, 44, 45]. This measurement is considered very crude and functional measurements on single fibres would be more informative. Studies examining the effects of myostatin deficiency on the contractile properties of dystrophic muscles have reported disparate results. Treatment with anti-myostatin antibody led to an increase in the maximal tetanic force generation, but when expressed as a function of muscle size specific force generation was decreased, whereas an improvement both in maximal and specific tetanic force is obtained when the propetide approach is used [19, 40]. These results obtained by the group of Bogdanovitch suggest that the effect on muscle force might be dependent upon the experimental approach. In addition, work from two independent laboratories has reported a decrease in force generation of muscles from myostatin null mutants [46, 47]. Earlier work from Mendias *et al. *[47] showed a decrease in specific force for edl muscle in myostatin knock-out mice whereas specific force of soleus muscle is unaffected. Likewise, Amthor and coworkers [46] reported that despite an increase in muscle mass in myostatin knock-out mice they observed no increase in maximal muscle force, but that specific muscle force was severely decreased both in edl and soleus muscles. Their results also revealed that such decrease in specific force is associated with mitochondrial depletion and loss of oxydative characteristics of skeletal muscle. Based on these observations it appears that excessive muscle growth following deficiency in myostatin compromises force generation. These results also suggest that muscle hypertrophy due to myostatin absence may in fact not be beneficial to skeletal muscle. In conclusion, these complexities underscore the importance of further studies of the effects of myostatin blockade before the results of experiments using antagonist drugs can be interpreted clearly. 

## MYOSTATIN AND SATELLITE CELLS

**Fig. (1):**
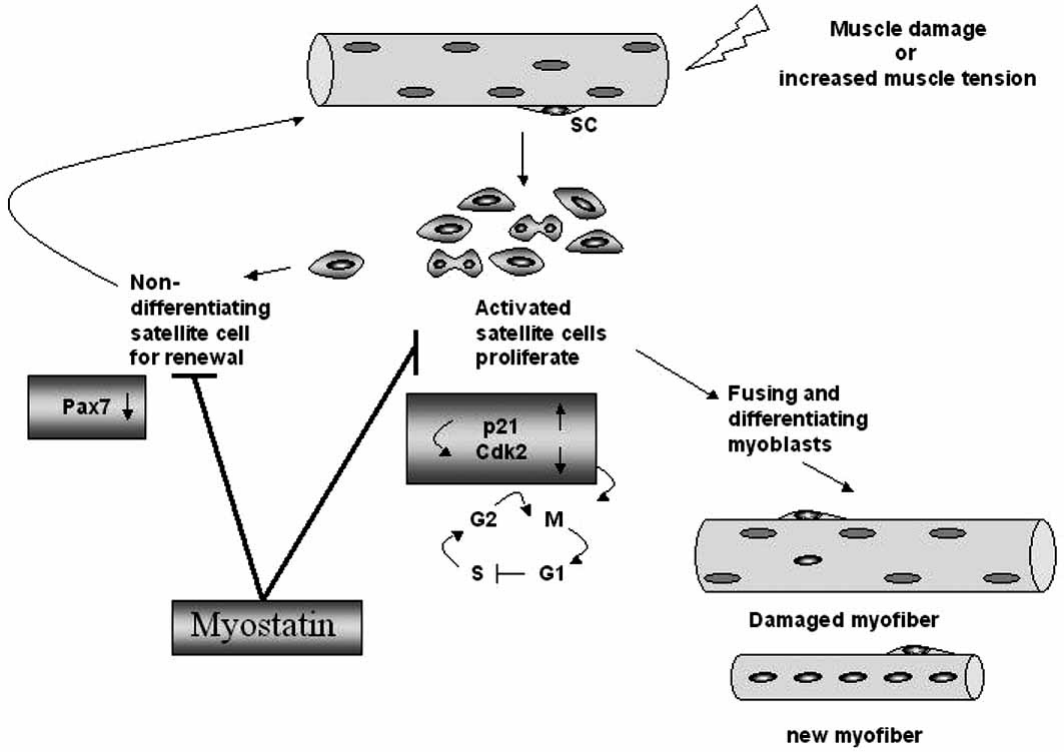
**The potential functions of myostatin in regulating satellite cells.**Growth and repair of adult skeletal muscle require the action of a population of normally quiescent myogenic precursors called satellite cells (SC) which reside between the basal lamina and the plasma membrane of the myofibres. In response to muscle injury or increased muscle tension, these cells become activated, start proliferating and are responsible for the repair of damaged muscle fibres and the growth of muscle fibres. Importantly, a proportion of the progeny of the activated satellite cells does not differentiate, but withdraws from the cell cycle to replace the activated satellite cell used, in a process of self-renewal. Myostatin negatively regulates satellite cell activation and self-renewal. A possible function is that Myostatin inhibits/prevents the progression of myoblats from the G1 to S phase of the cell cycle through up-regulation of p21 and subsequent inhibition of Cdk2 activity, thus maintaining the quiescence of satellite cells. Another function is that myostatin inhibits the self-renewal of the satellite cell population through down-regulation of Pax7.

Growth and repair of skeletal muscle are carried out by a group of quiescent muscle stem cells known as satellite cells. In response to tissue damage or hypertrophic stimuli, these cells are activated and will proliferate and differentiate [48-50]. However, a small proportion of these activated satellite cells does not differentiate, returns to quiescence to maintain the pool of satellite cells on the muscle fibers (Fig. **1**). Interestingly, myostatin has been proposed to be a key signalling molecule that signals the quiescence of satellite cells and their progeny, the myoblasts. Direct analysis of satellite cells in myostatin mutant mice has shown an increased number of satellite cells per single myofiber and a higher proportion of activated satellite cells compared to wild-type mice [51]. In addition, satellite cells isolated from myostatin null mice proliferate and differentiate more rapidly than satellite cells isolated from wild-type mice. These results suggest that myostatin maintains satellite cells in a quiescent state and that during regeneration or muscle growth these muscle progenitor cells could be reactivated upon release from the inhibitory effect of myostatin. In line with this notion, several groups have attempted to elucidate the intervention of myostatin in muscle regeneration after injury or in degenerative diseases [19, 45, 52]. It has been demonstrated that regeneration after injury in myostatin null muscle is improved compared to the wild-type controls. Recent evidence from McCroskery and coworkers [52] suggests that myostatin negatively regulates muscle regeneration not only by controlling satellite cell activation but also by regulating the migration of myoblasts and macrophages to the site of injury. Inhibition of myostatin in *mdx* mice that are known to have cycles of degeneration also reveals evidence for improved muscle regeneration in lack of myostatin [45]. The muscles of these mice show signs of persistent degeneration and regeneration, but they are bigger and exhibit an improvement of their histological features, such as decreased fibrosis compared to muscles of *mdx* mice [45]. Although the mechanism by which myostatin regulates muscle regeneration has yet to be clarified, these findings highlight the importance of myostatin in the process of muscle repair both after injury or in degenerative diseases. The importance of myostatin action in muscle repair is also substantiated by recent results of Sirriet *et al. *[53] showing that a short-term blockade of myostatin function significantly enhances muscle regeneration in aged mice after injury and during sarcopenia. This antagonism of myostatin led to satellite cell activation, increased expression of myogenic cell markers and greater myoblast and macrophage cell migration. These findings support earlier results showing that muscles of aged myostatin null mice continue to regenerate robustly from both chronic and acute injury [45, 54]. Therefore, the inhibition of myostatin appears protective against “normal” loss of muscle mass during aging. These result suggest that the absence of myostatin could also alleviate sarcopenia through the activation of satellite cells. In this respect, high serum concentrations of myostatin-immunoreactive protein have been found in older women and men with muscle wasting (sarcopenia) however, so far the role of myostatin in aging-induced sarcopenia has not been determined.

How does the myostatin signalling act on satellite cell activation, proliferation and differentiation? Recombinant myostatin is able to arrest the cell cycle at the G1 phase of cultured myoblasts including C2C12 myoblasts, primary bovine myoblats and mouse satellite cells, and thereby induces inhibition of proliferation and differentiation [3, 18, 51, 55]. It has been reported that myostatin can affect one or more of the cell cycle control proteins: the anti-proliferative action of myostatin is associated with an up-regulation of p21 (cyclin-dependent kinase inhibitor) and subsequently decreases in the levels of both cyclin-dependent kinases (Cdk2 and Cdk4) and phosphorylated Rb (retinoblastome protein) [3, 18, 21, 51] (Fig. **1**). Although the mechanism underlying the anti-proliferative action of myostatin has yet to be elucidated, but one possibility presented by Yang *et al. *[27] indicates that myostatin down-regulates Cdk4 activity *via *promotion of degradation of cyclin D1, an important component of the cell cycle machinery. Their results suggest that myostatin is capable of stimulating Akt inhibition, GSK-3β activation and cyclin D1 destabilization, and that this process is dependent upon the activin receptor IIB, but not Smad3. 

The inhibitory effect of myostatin on differentiation is associated with down regulation of the myogenic differentiation factors MyoD, Myf5 and myogenin [21]. Therefore the function of myostatin appears to derive from its inhibitory effects on the proliferation and differentiation of muscle cells.

Myostatin also inhibits satellite cell activation and self renewal [51]. Although the mechanism behind this function in satellite cell is not known, recent evidence from McFarlane *et al. *[56] suggests that myostatin signals *via *a Pax7-dependent mechanism to regulate satellite self-renewal (Fig. **1**). 

## ROLE OF MYOSTATIN IN MUSCLE ADAPTATION

The control of muscle mass is determined by a dynamic balance of anabolic and catabolic processes. Muscle hypertrophy is characterized by an increased in diameter and in total protein content of fibres and occurs as a result of an enhanced rate of protein synthesis while muscle atrophy induced by a decrease in activity and load or by catabolic loss of muscle mass is a decrease in the size of pre-existing muscle fibers, resulting from a dramatic increase in protein degradation and turn-over. A wealth of data supports that myostatin functions by inducing muscle atrophy. For example, elevated myostatin expression correlates with several settings of muscle loss including prolonged bed rest in young men [57], chronic disuse atrophy in older patients [58] and age-related muscle wasting (sarcopenia) [59]. Likewise, increased myostatin mRNA and protein expression are observed in a rat cirrhosis model that causes muscle atrophy [60].

Relevant to muscle wasting diseases, it has been shown that inhibition of myostatin rescued the muscular atrophy of caveolin-3-deficient mice [37]. The inhibition of myostatin also protects against muscle atrophy due to glucocorticoid treatment [61] and the effect of glutamine on prevention of glucocorticoid-induced skeletal muscle atrophy is associated with myostatin suppression [62]. Thus, induction of myostatin could explain the decreased protein synthesis observed in several settings of muscle atrophy. The crucial role of myostatin in inducing muscle atrophy is highlighted by the work from Zimmers *et al. *[6] demonstrating that administration of myostatin *in vivo* to adult mice produces the signs and symptoms characteristic of the muscle wasting syndrome, cachexia. In addition, the muscle wasting observed in these mice can be partially reversed by systemic delivery of the myostatin propeptide or follistatin in the mice indicating that the observed muscle wasting was caused by excess myostatin. Similarly, ectopic expression of myostatin through gene electrotransfer of a myostatin expression vector induces atrophy of skeletal adult muscle associated with decreased muscle gene expression [63]. The efforts of several laboratories have shed new insight into how myostatin induces muscle atrophy. Earlier work from Taylor *et al. *[55] showed that myostatin inhibited the proliferation as well as protein synthesis in C2C12 myoblasts and myotubes. Recently, McFarlane *et al. *[64] demonstrated that myostatin induces cachexia by activating the ubiquitin proteolytic system through a FOXO1-dependent mechanism. Importantly, it has been shown that FOXO1 also upregulates the expression of myostatin [65, 66]. Therefore, a positive feedback mechanism between these two pathways may amplify the atrophic response. While the above findings are undisputed it should be noted that these studies have involved overexpression of constitutively active myostatin which is likely exceed the strength of a physiological stimulus, resulting in possible loss of specificity. Future studies are therefore required to define whether chronic activation of myostatin is correlated with cancer cachexia. 

In contrast to the above findings, several independant studies have reported that myostatin might not be required in some types of muscle atrophy. Indeed, myostatin levels are not increased in muscle atrophy due to denervation or hindlimb suspension [67, 68]. Unexpectedly, the myostatin deficient mice lost more muscle mass than normal controls after hindlimb suspension [69]. These data indicate that absence of myostatin does not protect against muscle atrophy and also suggest that the action of myostatin might be to inhibit muscle hypertrophy rather than induce atrophy. These results also demonstrate that myostatin is likely not the primary cause of muscle atrophy. In conclusion, despite this progress in our understanding of the role of myostatin in muscle atrophy, mechanisms involved in regulation and actions of myostatin in all forms of muscle atrophy remain to be clarified. 

Another question that remains to be investigated is whether myostatin also plays a role in inactivating hypertrophic pathways. There is substantial evidence to suggest that myostatin could be connected with pathways regulating hypertrophy. The recent work of McFarlane [64] has shown that the protein kinase Akt is a target for myostatin action. Myostatin negatively regulates Akt by dephosphorylation of serine 473 which induces Foxo transcription factors and activates the expression of components of the ubiquitin-proteasome pathway such as MAFbx, to degrade muscle protein. Akt has been shown to be activated during hypertrophy, and it is a known upstream activator of protein synthesis [70, 71]. Therefore it appears possible that myostatin may also counter hypertrophic signalling through inactivation of Akt leading to decreased protein synthesis, but this has yet to be tested. Several data exist showing that myostatin appears to be sensitive to hypertrophic signals. For example, the anabolic agent GH decreased myostatin expression in muscles of GH-deficient adults [72] and androgens negatively regulate myostatin expression in an androgen-dependent skeletal muscle [73]. A similar down regulation of myostatin is observed in mouse muscles subjected to functional overload [74] and in human with strength-training exercise [75, 76]. Indeed, myostatin seems to play a role in modulating muscle mass in response of anabolic stimuli. These findings raise the question whether a negative feedback-loop exists between myostatin and hypertrophic pathways. Collectively these data suggest that atrophy and hypertrophy pathways could communicate with each other *via *myostatin. However, future researches should be focused on exploring how myostatin is connected with the cellular signalling pathways regulating the anabolic and catabolic responses in muscle. 

## CONCLUDING REMARKS AND KEY UNANSWERED QUESTIONS

Increasingly, research into the mechanisms by which myostatin controls muscle mass has revealed both new components of this signalling pathway and the participation of parallel pathways in the mediation of myostatin signal. It has been established that myostatin signalling can recruit various intracellular signalling cascades, however it remains unclear how these pathways are intercalated. With respect to new components of myostatin signalling pathway, decorin, a component of the extra-cellular matrix (ECM) has been discovered recently that binds myostatin and modulates its activity. This provides another means by which myostatin signalling can be controlled and raises the question of a potential link between myostatin activity and ECM integrity. Also, the demonstration that other TGF-β related ligands cooperate with myostatin to suppress muscle growth makes the search for manipulating TGF-β signalling pathways attractive.

It is well documented that myostatin is important for proper development and homeostasis of adult musculature. In this line, the link between myostatin and the Akt/Foxo pathway may account, at least in part, for observations that support a role for myostatin in breaking or promoting muscle wasting. It will be challenging and informative to determine whether myostatin can also interfere with anabolic pathways.

In the last decade, the blockade of myostatin has been represented an ideal tool to attenuate muscle wasting related to diseases, and there is a multi center clinical trial on adult patients with muscular dystrophy underway. The beneficial effects of loss of myostatin activity reside in enhancing muscle mass, however, there is also evidence that this increased muscle mass may in fact not be beneficial to skeletal muscle function. Indeed, Amthor *et al. *[46], recently reported that despite an increase in size the muscles of myostatin knock-out mice are weaker and show a predominance of fast glycolytic fibers, a loss in mitochondrial enzymes and a mitochondrial depletion. Further research will be necessary to balance the benefits and risks of myostatin blockade in the development of a beneficial therapy. It will be particularly important to elucidate the mechanisms of muscle metabolism that are affected by myostatin inhibition.

## Figures and Tables

**Table 1:** Summary of Data on the Effects of the Myostatin Binding Proteins

	*In Vitro*	Animal	Human
Myostatin propeptide	Antagonizes myostatin biological activity by inhibiting myostatin receptor binding [2]	Overexpression of propeptide associated with increased muscle mass [13, 14]	
FLRG	Inhibits myostatin activity in signalling assays, maintains myostatin latency [11]	Overexpression of FLRG associated with increased muscle mass [15]	
GASP 1	Inhibits myostatin activity in signalling assays, maintains myostatin latency [12]		
hSGT	Inhibits myostatin activity in signalling assays [10]		
Titin-cap (telethonin)	Myoblasts overexpressing titin-cap associated with increased myoblast proliferation, regulates myostatin secretion [9]		Lack of functional Titin-cap protein is responsible for a rare limb girdle muscular Dystrophy type 2G [17]
Follistatin	Inhibits myostatin receptor binding, antagonizes myostatin inhibition of myogenesis [7]	Overexpression of follistatin associated with increased muscle mass [13]; Loss of Follistatin associated with reduced muscle mass [16]	
Decorin	Blocks the inhibitory effect of myostatin on proliferation of muscle cells [8]		

## ABBREVIATIONS

TGF-β: = Transforming growth factor-β
GDF8: = Growth differentiation factor 8
BMP-1/TLD family: = Bone morphogenetic protein-1/tolloid family of proteinases
FLRG: = Follistatin like related gene
GASP: = Growth and differentiation factor-associated serum protein
SMADs: = Family of transcription factors that mediate TGF-β signals. The term SMAD is derived from the founding members of this family, the *Drosophila* protein MAD (Mothers Against Decapentaplegic) and the *Caenorhabditis elegans* protein SMA (Small body size).
MAPK: = Mitogen-activated protein kinase
AIDS: = Acquired immuno-deficiency syndrome
DMD: = Duchenne Muscular Dystrophy
ActRIIB: = Activin type II B receptor
GH: = Growth hormone
MAFbx: = Muscle atrophy F-box
